# Hayling and stroop tests tap dissociable deficits and network-level neural correlates

**DOI:** 10.1007/s00429-024-02767-7

**Published:** 2024-03-13

**Authors:** Margaret Jane Moore, Jessica Byrne, Emily C. Gibson, Lucy Ford, Gail A. Robinson

**Affiliations:** 1 Queensland Brain Institute, The University of Queensland, St Lucia, Brisbane, Australia; 2 Neuropsychology Research Unit, School of Psychology, The University of Queensland, St Lucia, Brisbane, QLD 4072 Australia

**Keywords:** Inhibition, Initiation, Strategy use, Lesion mapping

## Abstract

Although many executive function screens have been developed, it is not yet clear whether these assessments are equally effective in detecting post-stroke deficits of initiation and inhibition. This study presents a comparative analysis of the Stroop and Hayling tests aiming to evaluate whether these tests measure the same underlying cognitive functions and to identify the neural correlates of the deficits detected by both tasks. Sixty six stroke survivors and 70 healthy ageing controls completed the Hayling and Stroop tests. Stroke patients were found to exhibit qualitative performance differences across analogous Stroop and Hayling Test metrics intended to tap initiation and inhibition. The Stroop test was found to have high specificity to abnormal performance, but low sensitivity relative to the Hayling Test. Minimal overlap was present between the network-level correlates of analogous Stroop and Hayling Test metrics. Hayling Task strategy use metrics were significantly associated with distinct patterns of disconnection in stroke survivors, providing novel insight into the neural correlates of fine-grained behavioural patterns. Overall, these findings strongly suggest that the functions tapped by the Stroop and Hayling Test are both behaviourally and anatomically dissociable. The Hayling Test was found to offer improved sensitivity and detail relative to the Stroop test. This novel demonstration of the Hayling Test within the stroke population suggests that this task represents an effective measure for quantifying post-stroke initiation and inhibition deficits.

## Introduction

Executive dysfunction is a common and debilitating consequence of stroke affecting up to 50% of stroke survivors (Blake et al. 2002; Nys et al. 2006). The occurance of post-stroke executive function impairment acts as a robust predictor of poor long-term functional recovery outcomes and has a significant negative impact on stroke survivors’ ability to lead normal lives (Jefferson et al. 2006; Leśniak et al. 2008; Pohjasvaara et al. 2002). It is, therefore, critically important to reliably detect and diagnose post-stroke executive function deficits to inform prognoses and guide targeted rehabilitation. Although many standardised assessments of post-stroke executive function have been developed, it is not yet clear whether these assessments are equally effective in detecting specific aspects of executive dysfunction within the stroke population.

Broadly, executive function includes a range of cognitive abilities responsible for regulating, controlling, and managing other cognitive processes (Diamond 2013). Following stroke, executive dysfunction frequently manifests as an inability to effectively initiate or supress (i.e., inhibit) cognitive functions (Barker et al. 2017). Patients within initiation deficits struggle to begin actions (e.g., movements, verbal responses, activities), despite an intact understanding of when and how to do the correct actions (Zinn et al. 2007). Conversely, patients with suppression deficits struggle to stop or override dominant responses and have difficulties with interference control (selective attention/cognitive inhibition) and self-control (monitoring behaviour) (Chiappe et al. 2000; Diamond 2013; Guarino et al. 2020). Several standardised tools have been developed to assess initiation and suppression functions, but it is not yet clear whether each of these tools is equally effective within the stroke population.

First, the Stroop test (Stroop 1935) is commonly used to assess initiation and inhibition abilities within clinical populations. In this classical test, accuracy and response time are recorded as an individual reports the colour of non-word (e.g., dots) or word stimuli.

(Jensen and Rohwer 1966; Scarpina and Tagini 2017). Next, the person is asked to name the ink colour of colour words which are printed in incongruent colours (e.g., GREEN is printed in blue ink) (Jensen and Rohwer 1966). This task yields the control colour (non-word or word) response time as a measure of initiation and difference between this control and incongruent colour word conditions as a measure of suppression ability (Scarpina and Tagini 2017). Although this task is commonly used, it is not clear whether it serves as an optimal screen for post-stroke inhibition and suppression deficits.

By definition, to effectively assess cognitive inhibition (i.e., suppression), a task must involve the stopping of a response that has been initiated (Aron et al. 2014). It is unclear whether the Stroop test meets this criterium as successful completion of this task does not necessarily require subjects to supress an initiated response (Stuss and Alexander 2007). Cognitive interference from incongruent colour words cannot be clearly conceptualised as an “initiated response” as this information is task irrelevant and does not need to be considered for an appropriate response to be activated (Stuss and Alexander 2007). For example, subjects could adopt a strategy of naming the ink colour of single letters (e.g., the D in RED) to reduce interreference from incongruent colour words. As it is unclear whether incongruent responses are initiated in the Stroop test, it is unclear whether this task can adequately measure cognitive suppression abilities (Aron et al. 2014).

The Hayling Sentence Completion test (Burgess and Shallice 1996; 1997) is an alternative measure which has the potential to address many of the potential weaknesses of the Stroop test. In the first section of the Hayling test (sensible completion), participants are instructed to listen to 15 verbally presented sentence fragments and to verbally complete the sentences with a thematically related, sensible ending (e.g., The captain stayed with the sinking…*ship)*. In the second section (unconnected completion), participants are instructed to verbally complete 15 presented sentence fragments with completely unrelated endings (e.g., for the above example*…table)*. Critically, for subjects to complete this unconnected completion section, participants must successfully supress the automatic responses initiated by the semantic content of presented sentences (Aron et al. 2014). Accuracy on connected and unconnected response sections are compared to provide quantitative measurements of initiation and inhibition while qualitative response data can be used to provide insight into the strategies being used. Specifically, Hayling test responses can be classified into strategy type as identified by previous studies (Burgess and Shallice 1996; Robinson et al. 2015). For example, Burgess and Shallice (Burgess and Shallice 1996) observed that control participants tended to generate an unrelated word by naming visual objects around the room. Patients with frontal lobe damage have also been found to be less likely to implement strategies compared to healthy controls (Burgess and Shallice 1996; Robinson et al. 2015). Similar findings have been documented in older adults relative to younger adults. Hayling test strategy use metrics provide an additional and potentially valuable source of information which helps provide insight into the exact cognitive processes facilitating normal and abnormal initiation/inhibition (Gibson et al. 2018; Martin et al. 2021). However, the utility of the Hayling test has not yet been thoroughly investigated in the stroke population. Some previous studies have reported that stroke patients exhibit worse Hayling test performance relative to healthy controls (Laakso et al. 2019; Nijsse et al. 2019), but more fine-grained strategy and error type comparisons have not yet been reported. Therefore, the present study aims to address this knowledge gap.

Employing more precise diagnostic measurements of initiation can help further understanding of the neural correlates of these behaviours. While the neural correlates of general initiation and inhibition abilities have been investigated (Nathaniel-James et al. 1997), no previous investigation has conducted statistical lesion mapping analyses aiming to identify the correlates of Hayling task strategy use variables. Although initiation/inhibition deficits are traditionally associated with frontal lesions (Aron et al. 2014), recent work has suggested that different components of cognitive inhibition may be linked to separable frontal lobe correlates (Hung et al. 2018). Cipolotti and colleagues (Cipolotti et al. 2016) found that suppression on the Hayling test significantly relies on the right lateral prefrontal cortex (superior and middle frontal gyri), whereas the Stroop test was correlated with the left lateral superior and middle frontal gyri. This finding is in parallel with previous studies that link the right lateral frontal region with suppression on the Hayling test (Robinson et al. 2015) and left inferior frontal regions with the Stroop test (George et al. 1994; Taylor et al. 1997). This previous work provides preliminary evidence that the neural correlates associated with the Stroop and Hayling task may be independent, but more detailed analyses are needed to confirm this finding.

Complex functions such as inhibition and inhibition are not only supported by localised brain areas, but also rely on effective connection and communication between a distributed network of brain structures (Sadaghiani and Kleinschmidt 2016; Thiebaut de Schotten et al. 2008). Previous studies investigating the neural correlates of inhibition have employed mainly focal lesion location analyses (e.g., PET, ROI-level lesion overlay comparisons), and have not explored the role of network-level disconnections in these deficits (Robinson et al. 2015). While focal lesion approaches are important for identifying critical lesion sights, network analyses provide additional insight by quantifying how disruptions in communication between multiple spatially distinct regions is associated with neuropsychological deficits. Network-level lesion mapping is a powerful methodology for identifying statistically significant disconnection correlates of cognitive impairment (Gleichgerrcht et al. 2017). This method takes spatially distributed, disconnection-related effects into account when identifying brain-behaviour relationships (Foulon et al. 2018; Fox 2018; Gleichgerrcht et al. 2017). These methodologies consider probability of disconnection of each pre-defined network edge or tract as behavioural predictors rather than the presence/absence of damage on a voxel-by-voxel basis (Foulon et al. 2018). Network-based symptom mapping approaches have been applied to investigate the anatomy of many common post-stroke deficits but have not yet been applied to explore initiation and inhibition deficits. Therefore, this study aims to employ network-level lesion analyses to identify disconnection correlates of specific behavioural patterns on the Hayling and Stroop tests to elucidate the neural structures underlying common error patterns.

The present study aims to employ both the Hayling and Stroop test to assess initiation and inhibition functions in a large sample of healthy controls and stroke survivors. This study aims to provide a detailed investigation of the Hayling test in stroke survivors by conducting group-level comparisons of standard initiation/inhibition scores as well as more fine-grained comparisons of strategy use differences. This behavioural data will be used to conduct network-level lesion mapping analyses to identify and compare disconnection correlates of initiation/inhibition impairments as reported by the Stroop and Hayling tests. Overall, this study aims to provide a novel and detailed exploration of Hayling test performance and correlates in a large and representative sample of stroke survivors.

## Methods

### Participants

Sixty-nine acute stroke patients were recruited from the Royal Brisbane and Women’s Hospital and the Princess Alexandra Hospital (Brisbane, Australia). Patients were considered for inclusion in this investigation if they (1) were diagnosed with a first-time stroke as confirmed by routine clinical imaging (CT or MR) and (2) were within six weeks of hospital admission due to a stroke event. Patients were excluded from all conducted analyses if they exhibited evidence of previous strokes, were diagnosed with a Transient Ischaemic Attack, were > 90 years of age, were not fluent in English, and/or were affected by other psychiatric or neurological diagnoses (e.g., schizophrenia, substance use disorder, dementia). Participants were excluded from neuroimaging analyses (but included in behavioural analyses) if they lacked available neuroimaging data of suitable quality for the lesion analyses (e.g., poor quality scans). All participants provided informed written consent (in line with the Declaration of Helsinki) and approval for the study was provided by the Metro South and Metro North Health Human Research Ethics Committees and the University of Queensland (UQ) Human Research Ethics Committee.

Three patients were excluded from behavioural analyses due to having English as a second language (*n* = 2) or severe fatigue that precluded completion of most tasks (*n* = 1), whilst 12 participants were excluded from lesion-mapping analyses. Participant demographics and stroke descriptive statistics are reported in Table 1. Overall, 66 patients (mean age = 61.6 (SD = 13.9, range = 23–86), 33.3% female) were included in behavioural analyses and a subset of 54 (mean age = 60.5 (SD = 12.4, range = 23–84), 32.1% female) of these participants were included in lesion analyses. There was no significant differences in age (*t*(97.5) = 0.86, *p* = 0.39), gender (*X*^2^(1) = 0.06, *p* = 0.81), time between stroke and assessment (*t*(101.9) = 0.82, *p* = 0.41) or lateralisation of stroke (X^2^(3) = 0.20, *p* = 0.91) across these participant groups.

**Table 1:** Demographics for each of the patients included in this study

ID	Age	Sex	Handed	Education	Chronicity	Stroke type	Stroke side	Artery	Volume
P01	50	M	Right	12	19	Ischaemic	LH	MCA	61.1
P02	74	F	Right	10	19	Haemorrhagic	RH	–	
P03	69	M	Right	16	5	Ischaemic	RH	MCA	46.7
P04	59	M	Right	13	17	Ischaemic	LH	PICA	331.5
P05	63	M	Right	14	15	Ischaemic	RH	PCA	344.5
P06	84	F	Right	13	16	Ischaemic	RH	MCA	307.7
P07	28	F	Right	16	11	Ischaemic	LH	–	
P08	66	M	Right	12	16	Ischaemic	LH	Brainstem	8.0
P09	54	M	Right	15	39	Ischaemic	RH	MCA	183.5
P10	69	F	Left	17	21	Ischaemic	RH	MCA	81.7
P11	70	M	Right	15	25	Haemorrhagic	LH	MCA	236.8
P12	40	F	Right	16	48	Ischaemic	LH	MCA	10.4
P13	70	M	Right	10	62	Ischaemic	LH	PICA	23.7
P14	76	F	Right	14	26	Ischaemic	LH	PCA	654.0
P15	68	M	Right	10	46	Ischaemic	LH	PCA	524.2
P16	73	F	Right	16	46	Ischaemic	RH	MCA	0.09
P17	58	M	Right	11	45	Ischaemic	RH	MCA	17.4
P18	64	M	Right	10	26	Ischaemic	RH	MCA	225.8
P19	62	M	Right	18	24	Ischaemic	LH	PCA	
P20	67	M	Right	12	9	Ischaemic	RH	MCA/ICA	63.0
P21	72	F	Right	10	21	Ischaemic	LH	MCA	53.4
P22	50	M	Left	12	41	Ischaemic	RH	ACA	34.7
P23	86	F	Left	8	36	Ischaemic	LH	MCA	
P24	60	M	Right	15	21	Ischaemic	LH	MCA	19.8
P25	71	M	Right	20	26	Haemorrhagic	RH	ACA	103.7
P26	59	M	Right	16	14	Ischaemic	LH/RH	PICA	278.0
P27	86	M	Right	11	21	Ischaemic	RH	MCA	
P28	37	M	Right	12	8	Ischaemic	LH	MCA	413.2
P29	70	F	Right	16	27	Haemorrhagic	RH	MCA	545.4
P30	60	F	Right	14.5	35	Ischaemic	RH	MCA	42.9
P31	55	M	Left	12	26	Ischaemic	RH	–	
P32	81	F	Right	10	37	Ischaemic	RH	MCA	135.8
P33	55	F	Right	12	31	Ischaemic	LH	MCA	210.4
P34	41	M	Right	12	5	Ischaemic	RH/LH	PICA	180.9
P35	65	F	Right	12	32	Ischaemic	RH/LH	PICA	291.5
P36	64	M	Right	9	6	Ischaemic	RH	MCA	38.2
P37	57	M	Right	18	18	Ischaemic	RH	MCA	83.4
P38	51	M	Left	10	6	Ischaemic	LH	MCA	48.5
P39	65	M	Right	10	25	Ischaemic	RH	MCA	34.3
P40	70	F	Right	10	9	Ischaemic	RH/LH	PICA	107.3
P41	59	M	Right	10	6	Ischaemic	LH	MCA	236.8
P42	51	M	Left	10	5	Ischaemic	RH	MCA	31.3
P43	79	M	Right	8	4	Ischaemic	RH/LH	MCA	
P44	43	M	Right	12	6	Ischaemic	RH	MCA	80.3
P45	62	M	Right	7	3	Ischaemic	RH	ACA	9.6
P46	70	F	Right	12	6	Ischaemic	RH	–	10.0
P47	65	M	Right	10	9	Ischaemic	LH	PCA	343.3
P48	59	M	Right	12	10	Ischaemic	RH	Brainstem	31.7
P49	69	M	Right	13	13	Ischaemic	LH	MCA	
P50	45	F	Right	11	6	Ischaemic	RH	MCA/ICA	73.5
P51	61	M	Right	10	4	Ischaemic	RH	MCA	29.2
P52	74	M	Right	11	4	Ischaemic	RH	MCA	722.4
P53	81	M	Right	12	1	Ischaemic	RH	PCA	17.9
P54	61	M	Left	16	1	Haemorrhagic	LH	PCA	2.9
P55	76	M	Right	12	3	Ischaemic	RH	MCA	642.1
P56	58	M	Right	10	8	Ischaemic	RH	MCA	10.6
P57	48	F	Right	12	4	Ischaemic	LH	MCA	158.5
P58	68	M	Right	10	6	Ischaemic	LH	ICA	517.1
P59	68	M	Right	12	2	Ischaemic	RH	ACA	416.9
P60	49	M	Right	12	38	Ischaemic	RH	Lacunar	20.0
P61	41	F	Right	16	4	Ischaemic	RH	MCA	69.0
P62	23	F	Right	12	10	Ischaemic	LH/RH	MCA	1655.3
P63	43	F	Right	12	2	Ischaemic	LH	PCA	112.0
P64	69	F	Right	12	49	Ischaemic	RH	MCA	
P65	64	M	Right	9	32	Ischaemic	RH	Lacunar	
P66	60	M	Right	10	34	Ischaemic	RH	MCA	

Education is reported in years, Chronicity reports the number of days between stroke and assessment, Stroke Side reports the hemisphere impacted by stroke, and Artery reports the vascular territory impacted by each stroke (if known). Volume reports the lesion volume (in cm^3^) for the 54 patients who were included in this study’s lesion analyses

Behavioural data from 70 healthy ageing control subjects (mean age = 64.5 (SD = 10.3, range = 41–88), 42.8% Female, 5.7% left-handed) were also collected. These controls were recruited through the University of Queensland (UQ) networks under the above UQ Human Research Ethics. Controls were excluded if they reported a history of major neurological of psychiatric disorder. Control participants did not significantly differ from the included stroke participants in terms of age (*t*(123.6) =  − 1.42, *p* = 0.159, 95% CI =  − 6.90 to 1.41), sex (*X*^2^(2) = 0.958, *p* = 0.328 (Yates corrected)), or handedness (*X*^2^(2) = 1.054, *p* = 0.305 (Yates corrected)).

### Behavioural assessment

Each participant completed a series of standardised neuropsychological assessments aiming to determine pre-morbid cognitive abilities and to quantify executive function abilities. Specifically, each participant completed the National Adult Reading Test (NART) which, in line with standard protocols (Bright et al. 2002, 2018), was used to estimate pre-morbid optimal levels of functioning (Nelson and Willison 1991). In this task, participants are instructed to read a series of 50 increasingly difficult, irregularly spelled words aloud whilst a trained administrator scores each response for accuracy. This task is commonly employed in clinical environments to estimate pre-morbid intelligence (Nelson and Willison 1991).

Next, the Stroop Test (Victoria Version) was used to provide a standard measure of executive function abilities (Strauss et al. 2006). In this task, participants are first presented with 24 coloured dots arranged in a grid and are asked to name the colours of the dots as quickly as possible. Next, participants are presented with a grid of 24 non-colour words (e.g., FISH) printed in colour ink and are asked to name the ink colour of these stimuli as quickly as possible. Finally, participants are then presented with 24 colour words printed in an incongruent colour and are asked to name the ink colours as quickly as possible. According to standard guidelines, participants are scored according to the time required to complete each task, accuracy, and the interference time (time on the word condition divided by time on the colour dot condition) (Strauss et al. 2006).

Finally, each participant completed the Hayling Sentence Completion Test which was administered and scored in line with the published, standard guidelines (Burgess and Shallice 1997). Specifically, each participant was presented with 30 sentence frames which were missing the final word. In the first condition (*Initiation*) each sentence was read aloud and the participant was instructed to provide a word which completed each sentence frame in a logical and sensible manor (e.g., prompt = He posted a letter without a …, response = stamp). In the second condition (*Suppression*), this procedure was repeated but participants were instructed to complete each sentence with an unconnected response (e.g., prompt = He posted a letter without a…, response = banana). For each sentence, response time and response accuracy were recorded. These data were used to calculate average response time differences between Initiation and Suppression condition responses and to convert response time and accuracy scores into scaled scores (in line with standard scoring guidelines). Each scaled score ranged between 1 and 10, corresponding to the following normative data percentiles: 1 = out of normal range or < 1st percentile, 2 = 1st percentile, 3 = 5th percentile, 4 = 10th percentile, 5 = 25th percentile, 6 = 50th percentile, 7 = 75th percentile, 8 = 90th percentile, 9 = 95th percentile, 10 = 99th percentile (Burgess and Shallice 1997). All Suppression condition responses were categorised into eight possible response types (Robinson et al. 2015) (Table 2) to facilitate the analysis of strategy use. The frequency of each response category was recorded for each participant. The order of cognitive tests was counterbalanced to prevent systematic variance due to testing order.

**Table 2:** Categories used to categorise responses from the Hayling Test Suppression condition. These categories were defined by Robinson et al. (Robinson et al. 2015) based on the data reported by Burgess and Shallice (Burgess and Shallice 1996)

Response category definition	Description and examples
Category A error (Blatant connected response)	
A1. Sensible sentence completion	“The dough was put in the hot…*oven*”
Category B error (Partially connected response)	
B2. Semantically related/opposite to response B3. Semantically related to sentence B4. Semantically related and bizarre	“The dough was put in the hot…*sink/freezer*” “The dough was put in the hot…*bread*” “The whole town came to hear the mayor…*cry*”
Category C correct response (unconnected to sentence)	
C5. Correct and visible C6. Correct and semantically related to previous response C7. Correct and both visible and semantically related to previous response C8. Correct with no obvious strategy C9. Correct with other strategy	“The dough was put in the hot…*table*” “The dough was put in the hot…*orange*” (previous response* “banana”)* “The dough was put in the hot…*chair*” (previous response “*table*”) “The dough was put in the hot…*train*”

### Neuroimaging data

**Fig. 1:**
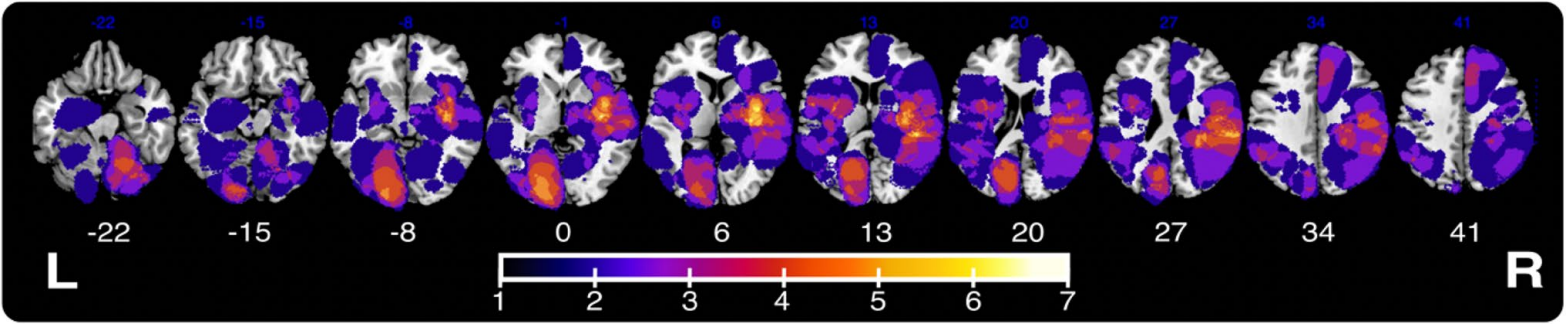
Lesion overlay for the 54 patients included in this study’s lesion analyses. Colour represents the number of lesions overlapping at each location. MNI z-coordinates -22–41 are visualised

Routinely collected clinical neuroimaging data (10 CT, 1 T1 MR, 43 FLAIR) was employed to create binarized lesion masks for each included patient (Fig. 1). Previous research has demonstrated that similar routinely collected neuroimaging data are of sufficient quality to reliably create lesion masks which are able to accurately identify the correlates of a wide range of cognitive impairments in acute stroke populations (Moore et al. 2023a, b, c; Moore and Demeyere 2022). Further, Moore et al. (2023a, b, c) have demonstrated that lesion mapping results yielded from CT-based analyses agree well with those produced using MR modalities. Within this study, all lesion masks were processed using the standard processing protocol reported by Moore (2022). Specifically, all lesions were manually delineated on native space scans by trained experts and smoothed at 5 mm full width at half maximum in the z-direction using MRIcron (Rorden 2007). These native-space masks and scans were then reoriented to the anterior commissure and warped into 1 × 1x1 MNI space using Statistical Parametric Mapping (Ashburner et al. 2016) and age-matched templates from Clinical Toolbox (Rorden et al. 2012). All normalised scans were visually inspected for quality prior to inclusion in lesion mapping analyses.

### Lesion mapping analyses

As previous research has strongly suggested that initiation and inhibition require collaboration between a distributed network of neural correlates, network-level lesion mapping analyses were employed in this study. This technique is commonly employed and offers key insight into how disruptions in brain networks are associated with cognitive impairments (Cohen et al. 2021; Moore et al. 2023a, b, c; Saxena et al. 2022).

First, lesion quantification toolkit (Griffis et al. 2021) was used to generate disconnection statistics by overlaying each binarised lesion masks onto the Schaefer-Yeo Atlas Parcellation (100 parcels, 7 Networks) (Schaefer et al. 2018). An additional 35 subcortical ROIs derived from the Harvard–Oxford Subcortical Atlas and the Automated Anatomical Labelling Atlas (version 3). These atlases were employed as, together, they offer an optimal balance between complexity and interpretability. Lesion quantification toolkit (Griffis et al. 2021) estimates parcel-wise disconnection severities by calculating the number of normative HCP-842 streamlines which bilaterally terminate within each pair of atlas-defined grey matter parcels. This process yields disconnection matrices in which the value in each cell represents the percentage of disconnected streamlines (edges) connecting each of the defined grey matter parcels (nodes) per patient (135 nodes, 18,225 edges) (Griffis et al. 2021). For each network edge a regression was conducted to compare percent disconnection to behavioural scores. Each regression controlled for lesion volume, and only edges damaged in > 10% of included patients were analysed (1766 edges included). All conducted comparisons employ 5% false discovery rate to correct for inflated false positive rates due to multiple comparisons. This approach is similar to network-level lesion-mapping methodologies employed in previous studies (Gleichgerrcht et al. 2017; Saxena et al. 2022).

In total, 21 network-level lesion mapping analyses were conducted using the following outcome variables: Stroop Dot test (mean RT), Stroop colour-word test (mean RT, interference score), Hayling Initiation (mean RT, total Correct), Hayling Inhibition (mean RT, total correct), Hayling Strategy use (A1-C8, no obvious strategy count, proportion of responses using strategies), Hayling Scaled Score (calculated in line with standard scoring procedures), Hayling difference between Initiation/Suppression response times, and Hayling Global Error Score (total number of errors).

In addition to this, network-level overlap comparisons were conducted to evaluate the degree of similarity between the correlates associated with Stroop and Hayling Test scores aiming to assess analogous functions. Specifically, three overlap comparisons were conducted to compare Stroop Dot Time score versus Hayling Initiation time, and Stroop Colour-Word Time versus Hayling Inhibition time, Stroop Interference Score versus Hayling Inhibition versus Initiation time difference. In each case, the number and identity of any network edges impacted in both compared tests are reported.

### Statistical analyses

First, included behavioural data were inspected for statistical outliers which deviated from score means by > 3.5 standard deviations. This more liberal inclusion threshold was adopted to account for the large amount of behavioural variance expected within the acute stroke population (Demeyere et al. 2015). As the majority of considered behavioural variables were not normally distributed (*p* < 0.05), non-parametric tests were used (i.e., Spearman’s correlations and Mann–Whitney *U* tests for between-group comparisons). All reported confidence intervals (CI) refer to 95% confidence intervals.

Next, differences in demographic and clinical characteristics were examined between the acute stroke and healthy control groups. Specifically, Mann–Whitney *U* tests were used for continuous variables (i.e., age, years of education, estimated premorbid intelligence) and Chi-square tests for independence (with Yates continuity correction) were used for categorical variables (i.e., biological sex and handedness).

To identify significant Stroop and Hayling test performance differences between control and stroke patients, Mann–Whitney *U* tests were conducted on each of the 21 outcome variables of interest (Stroop Dot test (mean RT), Stroop colour-word test (mean RT, interference score), Hayling Initiation (mean RT, total Correct), Hayling Inhibition (mean RT, total correct), Hayling Strategy use (A1-C8, no obvious strategy count, agrammatical correct responses count, proportion of responses using strategies), Hayling Scaled Score (calculated in line with standard scoring procedures), Hayling difference between Initiation/Suppression response times), and Hayling Global Error Score (total number of errors).

Within strategy use variables, prevalence of each response type was calculated as a proportion of either correct or incorrect responses. For example, incorrect answers (categories A and B) were calculated by dividing the subcategory raw score by the total number of errors. Conversely, prevalence of correct answer types (Category C) was calculated by dividing the subcategory raw score by the total number correct. This strategy was adopted to ensure these analyses were investigating proportion of reliance on each strategy/error type independent of overall accuracy. Where appropriate, Bonferroni corrections for multiple comparisons are employed. Each applied alpha threshold is reported and justified prior to reporting results of each conducted comparison.

Performance on analogous Stroop and Hayling Test variables was compared both in terms of score correlations (non-parametric Spearman rank correlations) and in terms of sensitivity/specificity. These sensitivity/specificity calculations are not meant to be interpreted in terms of diagnostic accuracy but are instead reported to comprehensively summarise the degree of agreement between normal/abnormal performance categorisations reported by each of these tasks. These comparisons report the sensitivity/specificity of Stroop Test metrics relative to the “standard” of Hayling test metrics.

As past literature has suggested that Category B Hayling errors are subtle semantically related responses rather than blatant inhibition failures, the underlying cognitive process may differ from inhibition per se. In focal frontal lesion patients, all frontal patients make a high number of Category A Hayling errors (i.e., blatant inhibitory failures) but only the right lateral frontal patients make a high number of Category B Hayling errors, which are subtle and likely implicate a failure of other processes such as monitoring (Robinson et al. 2015, 2016). Further, healthy older adults increasingly produce category B but not A errors (Gibson et al. 2018). Taken together, these findings suggest differing cognitive and neural underpinnings. A series of secondary, exploratory analyses were, therefore, conducted to identify patterns of disconnection associated with these errors in more detail. First, linear regression and ANOVA analyses were conducted to identify stroke-related and clinical factors associate with the occurrence of Category B Hayling errors. These analyses included age, stroke side, stroke territory, and lesion volume as predictors. Next, lesion overlap images were created to visualise qualitative difference between patients in the top and bottom quantiles of Hayling Category B error commission. A similar analysis was repeated within the network-level data to identify connections that, when disrupted, resulted in high versus low occurrence of Category B errors. Specifically, the value of each considered edge was determined by calculating the number of Category B errors which occurred in patients with damage to the edge and dividing this by the number of patients with damage to this edge. This score summarizes the prevalence of Category B errors at each edge whilst accounting that some edges were damaged more frequently in the sample than others. Importantly, these visualisations are not meant to identify statistically significant correlates, but instead aim to identify potential trends which can then be further investigated in larger samples.

## Results

### Behavioural results

Stroke patients were found to commit significantly more errors on the NART than controls (patient mean = 22.9 (SD = 7.05, range = 3–42) versus control mean = 16.6 (SD = 6.94, range = 4–36) (W = 2968.5, *p* < 0.001, 95% CI 5.00–10.00)). In line with this, controls had a higher average estimated IQ than stroke patients (patient mean = 102 (SD = 8.73, range = 79–127) versus control mean = 110 (SD = 8.65, range = 86–126))(W = 953.5, *p* < 0.001, 95% CI: − 12.0 to − 6.0).

Next, control and stroke patient Stroop Test performance was compared. As 4 Stroop Test variables were compared across groups, a Bonferroni-corrected alpha threshold of 0.0125 was used for these analyses. Patients took significantly more time than controls to complete the Stroop test Dot condition (patient mean = 16.6 s, control mean = 12.5 s, *W* = 2766, *p* < 0.001, CI 1.81–4.37). Patients and controls completion times were also significantly different within the Stroop Colour-Word condition (patient mean = 35.7 s, control mean = 26.5 s, W = 2486, *p* < 0.001, CI 2.45–9.07). However, Stroop Colour-Word Interference scores were not significantly different between patient and control subjects (patient mean = 2.25, control mean = 2.15, *W* = 1830.5, *p* = 0.8505, CI − 0.17 to 0.22) and patients and controls were not found to have significantly different Stroop Colour-Word accuracy scores (patient mean = 64.61% correct, control mean = 75.5% correct, *W* = 1412, *p* = 0.0176, CI − 2.40 to − 1.9 × 10^–5^).

**Fig. 2:**
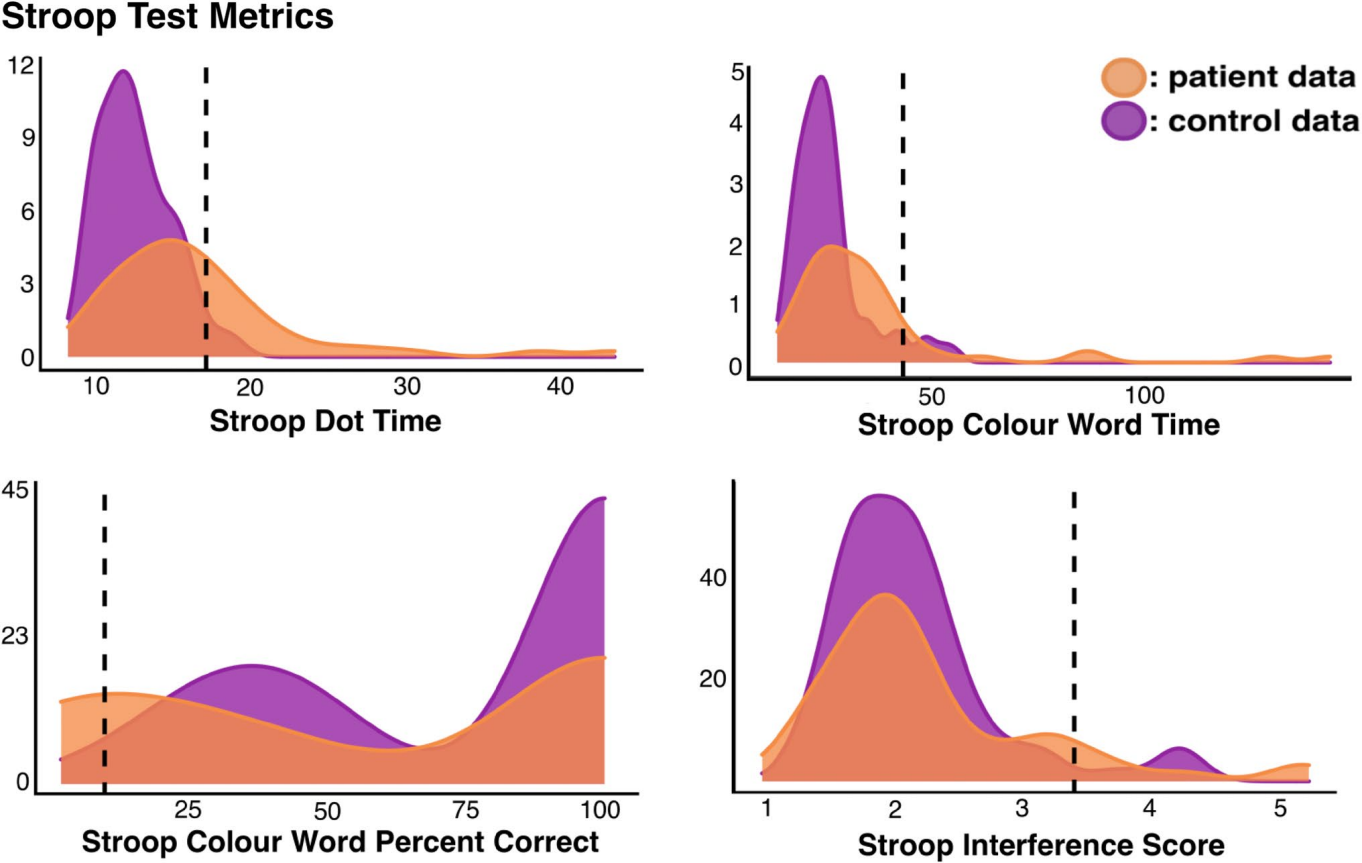
A visualisation of control and patient performance on the Stroop Test. Number of participants is represented on the y-axis and impairment cut-off thresholds are marked by dotted lines

Control data were then used to calculate Stroop Test impairment thresholds and identify patients exhibiting abnormal performance (Fig. 2). Stroop Dot times > 17.5 s, Colour-Word times greater than 43.6 s, interference scores > 3.40 s, and cumulative percent correct scores < 9.86 were considered to represent abnormal performance. In line with these thresholds, 18 patients exhibited abnormal scores on Stroop Dot Time, 8 on Colour-Word Time, 12 on Colour-Word Percent Correct, and 5 on Interference Scores.

Next, control and participant Hayling Test performance was compared. As seven independent Hayling performance metrics were included in these analyses, a Bonferroni corrected alpha level of 0.006 was employed. Table 3 summarizes the results of these comparisons. Hayling Overall Scaled Score was found to be significantly lower within patients than within control participants. Correspondingly, Hayling Global Error score was significantly higher in patients than in controls. Hayling Initiation Total Correct Scores were not found to be significantly different between patients and controls, but patients exhibited significantly lower Hayling Inhibition Total Scores than controls. Control participants were found to employ strategies in a significantly higher proportion of correct response trials. All other considered that general Hayling performance variables were not found to differ significantly across patients and controls (see Table 3 for details).

**Table 3:** A comparison of patient versus control group performance on the Stroop and Hayling Test general performance metrics

Metric	Patient	Control	*W*	*p*	CI	Cut	Nimp
Stroop test							
Dot time	**16.64 (6.6)**	**12.5 (2.3)**	**2766.0**	** < 0.001**	**1.81** to **4.37**	** > 17.05**	**18**
Colour-word time	**36.84 (24.4)**	**26.5 (8.6)**	**2486.0**	** < 0.001**	**2.45** to **9.07**	** > 43.6**	**8**
Colour word percent correct	58.64 (75.5)	75.5 (32.8)	1412.0	0.018	− 24.00 to 0.00	< 9.86	12
Interference score	2.27 (0.9)	2.15 (0.64)	1830.5	0.850	− 0.17 to 0.22	> 3.4	5
Hayling test							
Overall scaled score	**4.21 (1.8)**	**5.63 (1.28)**	**1156.0**	** < 0.001**	**− 2.00** to **0.00**	** < 3.1**	**21**
Global error score	**12.69 (11.9)**	**5.6 (5.36)**	**2747.5**	** < 0.001**	**1.00** to **7.00**	** > 16.3**	**20**
Proportion strategy use	**0.43 (0.3)**	**0.63 (0.25)**	**1294.0**	** < 0.001**	**− 0.30** to − **0.08**	** < 0.13**	**12**
Initiation mean RT	1.31 (0.9)	0.94 (0.39)	2310.5	0.031	0.02–0.33	> 1.72	10
Initiation mean correct	14.35 (1.5)	14.8 (0.55)	1749.0	0.109	0.00–0.00	< 13.6	7
Inhibition mean RT	4.43 (3.8)	2.86(1.96)	2384.0	0.012	0.21–1.61	> 6.8	10
Inhibition/initiation RT difference	47.6 (66.0)	29.1 (28)	2298.5	0.199	− 3.00 to 18.00	> 85.1	14
Inhibition total correct	**8.5 (4.5)**	**11.4 (3.01)**	**1248.0**	** < 0.001**	**− 4.00** to **− 1.00**	** < 4.7**	**18**

Group means are presented alongside standard deviations (in parentheses)

*P* p-value, *CI* 95% confidence interval boundaries, *max cut* control mean plus two standard deviations, *min cut* control mean minus two standard deviations, *N imp* number of patients with scores outside this normative performance range

**Fig. 3:**
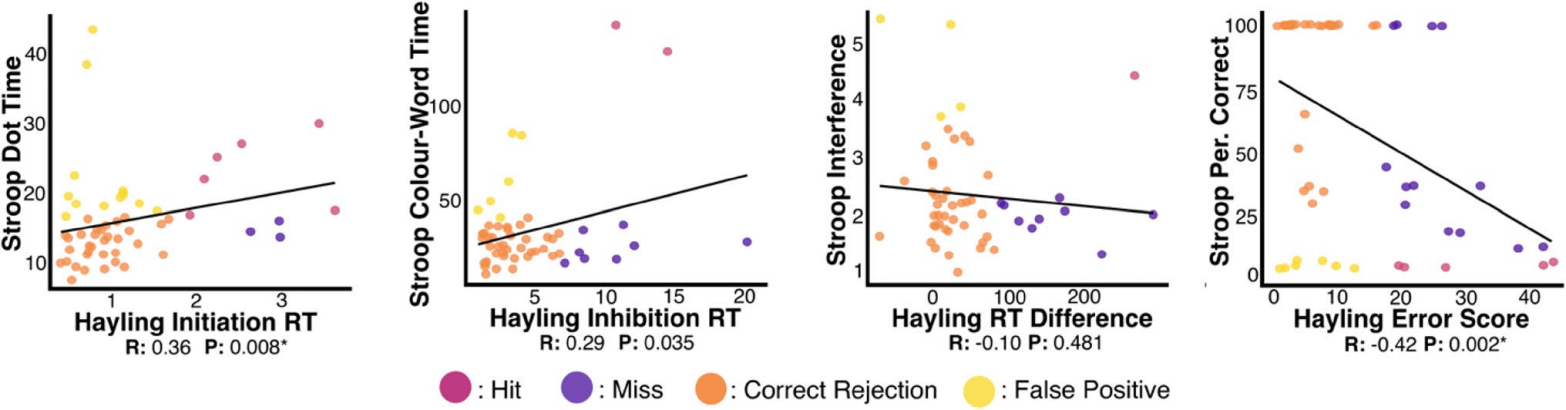
A comparison of analogous metrics derived from Stroop and Hayling Tests. Outcome categories are calculated using Hayling Scores as references. R reports rho values from Spearman’s correlations. Comparisons surviving multiple corrections are starred

Notably, significant disparity was present within the specific patients who met abnormal performance thresholds on analogous Stroop and Hayling Test metrics. Only 6/18 of the patients exhibiting abnormal performance on the Stroop Dot Time metric also exhibited abnormal performance on Hayling Initiation Time (sensitivity = 66.6%, specificity = 75.0%). Of the 8 patients exhibiting impairment on the Stroop Colour-Word Time metric, 2 were also impaired on Hayling Inhibition Response Time (sensitivity = 20.0%, specificity = 86.0%). Of the 5 patients with abnormal Stroop Interference Scores, 1 exhibited abnormal performance on the Hayling Response Time Difference Metric (sensitivity = 10.0%, specificity = 90.5%). Finally, 6/12 patients with abnormal Stroop Test Percent Correct also exhibited abnormal performance according to the Hayling Global Error Score (sensitivity = 27.8%, specificity = 80.0%). In addition to this, regression analyses revealed that scores analogous Stroop and Hayling metrics measuring initiation time and overall percent correct were significantly correlated, but analogous scores aiming to tap inhibition time and interference were not significantly correlated (Bonferroni-corrected alpha = 0.0125, see Fig. 3).

Next, differences in Hayling Strategy use were compared across patient and control groups. As 10 distinct error/strategy categories were considered, a Bonferroni-corrected significance threshold of 0.005 was used within these analyses (Table 4). Within incorrect responses, patients were found to commit a greater proportion of errors in which the response was semantically related (B3) to a sensible completion. Within correct responses, controls exhibited a greater proportion of responses using visible objects (C5) and visible objects which were related to previous responses (C7). Conversely, stroke patients exhibited a higher proportion of correct responses which did not use a strategy than controls (C8). All other considered variables were not found to be significantly different across groups (Table 4).

**Table 4:** A comparison of patient versus control group performance on Hayling Test error type and strategy use metrics

Strategy use variables								
Category	Patient	Control	*W*	*p*	CI	Max cut	Min cut	Nimp
A: Blatant connected	0.35 (0.3)	0.26 (0.3)	1638.5	0.0530	**− **0.20 to 0.00	0.92	**− **0.40	5
B: Partially connected	0.6 (0.3)	0.54 (0.4)	1942	0.6710	**− **0.17 to 0.07	1.36	**− **0.28	0
B2: Related/opposite	0.25 (0.3)	0.28 (0.3)	2066.5	0.8590	**− **0.03 to 0.06	0.90	**− **0.34	2
B3: Related	**0.2 (0.3)**	**0.08 (0.2)**	**1511.5**	**0.0040**	**− 0.08** to **0.00**	**0.41**	**− 0.26**	**11**
B4: Related/bizarre	0.15 (0.2)	0.18 (0.2)	2181	0.4460	0.00 to 0.06	0.66	**− **0.29	3
C5: Visible	**0.2 (0.3)**	**0.34 (0.2)**	**2779.5**	** < 0.001**	**0.08** to **0.22**	**0.82**	**− 0.13**	**2**
C6: Previous response	0.16 (0.2)	0.16 (0.1)	2295.5	0.1950	0.00 to 0.08	0.45	**− **0.12	7
C7: Visible and related	**0.05 (0.1)**	**0.1 (0.1)**	**2692**	** < 0.001**	**0.00** to **0.08**	**0.34**	**− 0.14**	**2**
C8: No strategy	**0.55 (0.3)**	**0.37 (0.3)**	**1351.5**	**0.0012**	**− 0.29** to **− 0.07**	**0.87**	**− 0.12**	**11**
C9: Other strategy	0.03 (0.1)	0.02 (0.08)	1915	0.3400	0.00 to 0.00	0.17	**− **0.14	2

Group means are presented alongside standard deviations (in parentheses)

*P p-*value, *CI* 95% confidence interval boundaries, *max cut* control mean plus two standard deviations, *min cut* control mean minus two standard deviations, *N imp* number of patients with scores outside this normative performance range

### Lesion-mapping results

**Fig. 4:**
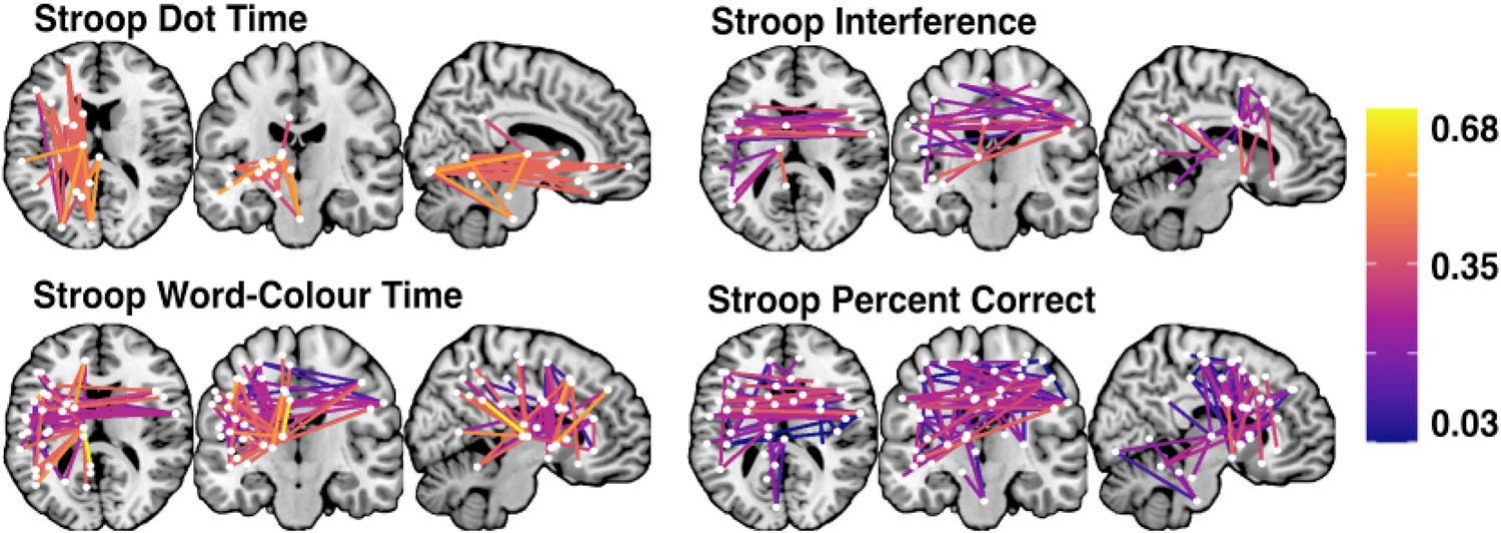
Schaefer-Yeo Atlas network edges significantly associated with Stroop Test metric. Nodes are plotted as white dots and edge colour represents adjusted *R*^2^ value for each base regression analysis. On each slice, node locations are collapsed into 2 dimensions and plotted in order of ascending *R*^2^ value (highest *R*^2^ values on top). Names, MNI coordinates, and statistics for each significant node are openly available https://osf.io/mbsd4/

Next, network-level lesion mapping analyses were conducted to identify patterns of dysconnectivity significantly associated with performance on each considered behavioural metric. Full lists of the network edges associated with each considered outcome measure can be found in supplementary materials. Within the Stroop Test, response time within the dot condition was significantly associated with dysconnectivity in 27 edges spanning left hemisphere temporo-parietal networks including the visual, default, and limbic networks as well as several subcortical nodes (lenticular and caudate nuclei) (Fig. 4). Response time within the Stroop Colour-Word Interference condition was also associated with dysconnectivity within left hemisphere temporo-parietal networks, but was also linked to disconnection within cross-hemisphere connections linking left and right hemisphere fronto-temporal areas. Accuracy within the Stroop Colour-Word Interference condition was primarily associated with damage to these cross-hemisphere fronto-parietal network connections. Finally, overall percent correct on the Stroop Test was associated with disconnection within left hemisphere temporo-parietal areas as well as some cross-hemisphere structural connections (Fig. 4).

**Fig. 5:**
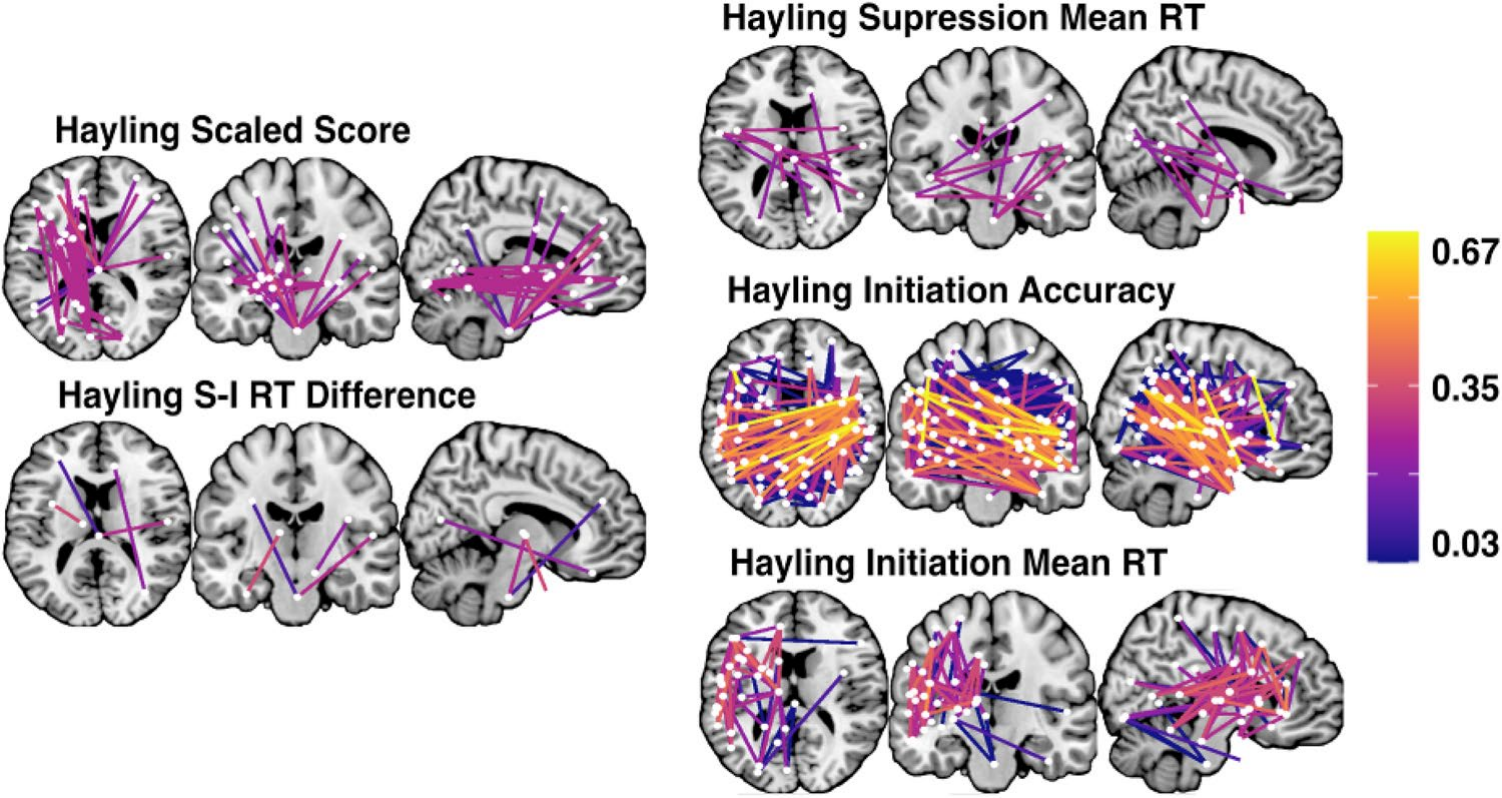
Schaefer-Yeo Atlas network edges significantly associated with Hayling Test general performance metrics. Nodes are plotted as white dots and edge colour represents adjusted *R*^2^ value for each base regression analysis. On each slice, node locations are collapsed into 2 dimensions and plotted in order of ascending *R*^2^ value (highest *R*^2^ values on top). Names, MNI coordinates, and statistics for each significant edge are openly available https://osf.io/mbsd4/

Hayling Overall Scaled Score was associated with disconnection within 66 network edges, mainly involving dysconnectivity between left hemisphere posterior and frontal areas as well as disconnection between right hemisphere frontal areas and the brainstem. Accuracy within the Initiation Condition was linked to disconnection within a large number of network edges (*n* = 302) spanning a diverse range of correlates in both the right and left hemispheres. Notably, no network edges were found to be associated with number of correct responses within the Hayling Suppression Condition. Mean response time within the Hayling Initiation Condition was associated with damage to 59 network edges spanning left hemisphere language areas and cross-hemisphere fronto-temporal connections. Conversely, mean response time within the Suppression condition was linked to damage to 11 edges mainly linking left hemisphere ventral attention network nodes to right hemisphere dorsal attention, limbic, visual, and default network nodes. Finally, response time difference between the suppression and initiation conditions was associated with disconnection within 6 edges, connecting a diverse range of right and left network nodes (Fig. 5).

**Fig. 6:**
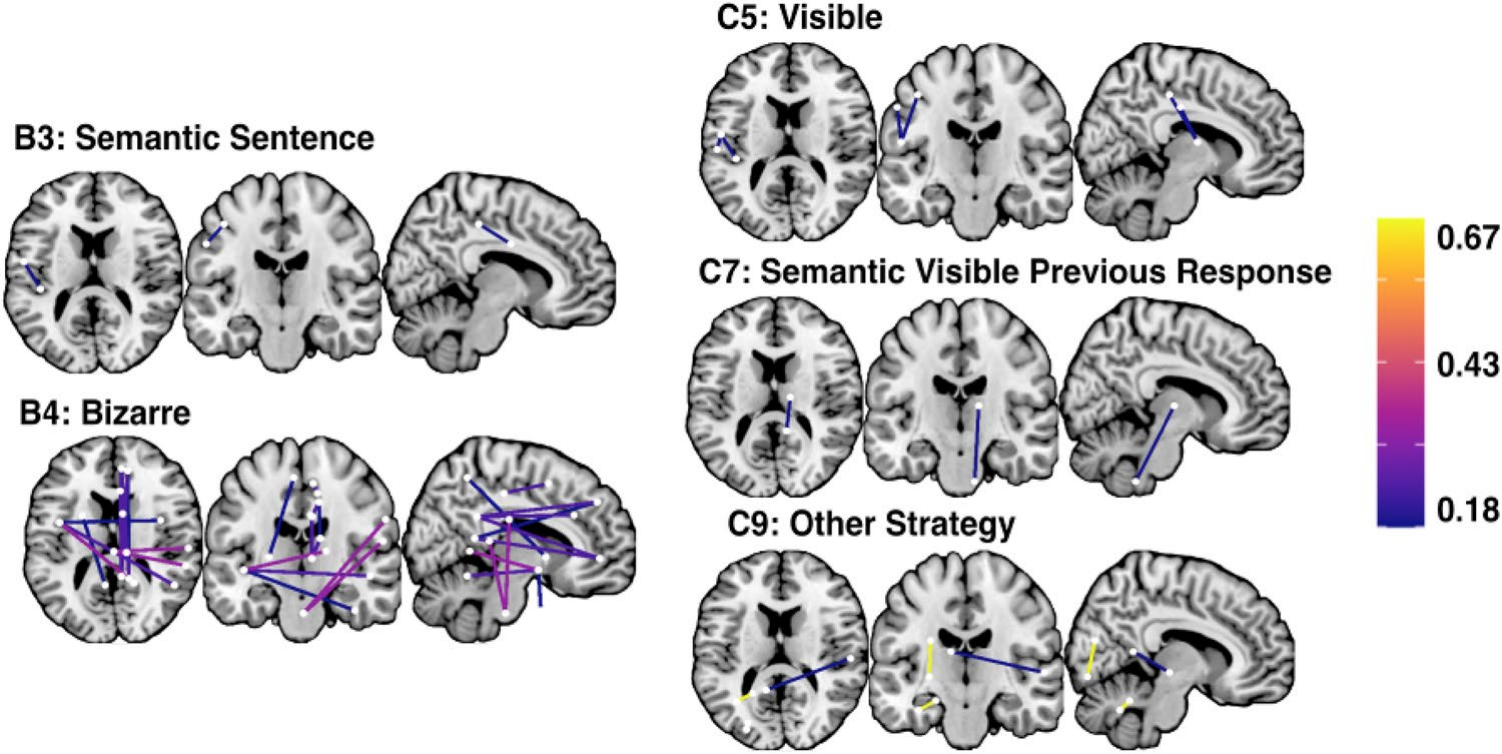
Schaefer-Yeo Atlas network edges significantly associated with Hayling Test strategy use metrics. Nodes are plotted as white dots and edge colour represents adjusted *R*^2^ value for each base regression analysis. On each slice, node locations are collapsed into 2 dimensions and plotted in order of ascending *R*^2^ value (highest *R*^2^ values on top). Names, MNI coordinates, and statistics for each significant node are openly available https://osf.io/mbsd4/

Finally, five of the considered ten Hayling Strategy use variables were significantly associated with localised patterns of structural dysconnectivity (Fig. 6). Inhibition section errors involving errantly reporting a response associated with the sentence (Category B3) were linked with disconnection in a single edge connecting the left hemisphere somatic motor network (division 4) to the left dorsal attention network posterior division 4. Errors involving bizarre strategies (B4) were associated with disconnection in 12 network edges connecting a range of right and left hemisphere structures. Inhibition section correct responses involving reporting an unrelated, but visible object response were linked to disconnection within two edges connecting the left hemisphere somatic motor network (division 3) to the left dorsal attention network (posterior divisions 2 and 4). Correct responses which were semantically connected to previous responses and visible (category C7) were associated an edge connecting the right caudate nucleus to the right cerebellum (division 9). Finally, correct answers employing other strategies were linked to disconnection in three edges connecting the left visual network divisions 4 and 8, the left default network precuneus/posterior cerebral cortex (division 1) and the right default temporal division 3, and connecting the left cerebellum (crus) to the left cerebellum (division 6).

### Comparison of stroop and hayling neural correlates

Four network-level overlap comparisons were conducted to evaluate the degree of similarity between the correlates associated with Stroop and Hayling Test scores aiming to assess analogous functions. First, the network level correlates of Stroop Dot Time and Hayling Initiation response time were compared. Of the 86 network-edges found to be significantly related to these variables, 6 were common across both tests. These common edges mainly involved connections between left hemisphere visual areas (divisions 4 and 5) and the caudate nucleus and brainstem. The network edge connecting the left caudate nucleus and left default network precuneus/posterior parietal cortex component was also significantly associated with performance on both tests.

Next, networks associated with Stroop Colour-Word time and Hayling Inhibition time were compared. Of the 78 edges associated with these conditions, only one connection between the left caudate nucleus and left default network (posterior parietal/precuneus division 2) was common across both tests. No common edges were present between the networks associated with Stroop Interference Scores and Hayling Inhibition versus Initiation response time difference.

### Secondary analyses: neural basis of category B hayling errors

Finally, a series of exploratory analyses were conducted to identify neural correlates associated with the commission of category. Within patients, the proportion of errors classed as Category B was not found to be significantly predicted by stroke volume (*F*(1,50) = 1.197, adjusted *R*^2^ = 0.004, *p* = 0.2791) but was predicted by age (*F*(1,54) = 5.552, Adjusted *R*^2^ = 0.07644, *p* = 0.022) (Bonferroni-corrected alpha = 0.05). Patients with left hemisphere strokes committed a significantly higher proportion of Category B errors than patient with right hemisphere strokes (0.742 vs 0.525 respectively, *F*(1,53) = 7.61, *p* = 0.008). However, this proportion was not significantly different across patients with MCA (0.535), ACA (0.543), PCA (0.751), lacunar (0.641), and PICA (0.639) territory stokes (*F*(4,46) = 0.943, *p* = 0.448). Notably, when all these considered factors were entered into a single regression analysis, no factors were found to be significantly predictive of the proportion of Category B errors committed (all *p* values > 0.60). This relationship remained unchanged when the raw number of Category B errors committed (instead of proportion of Category B errors) was used as the outcome variable (all *p* values > 0.027, Bonferroni-corrected alpha = 0.006).

**Fig. 7:**
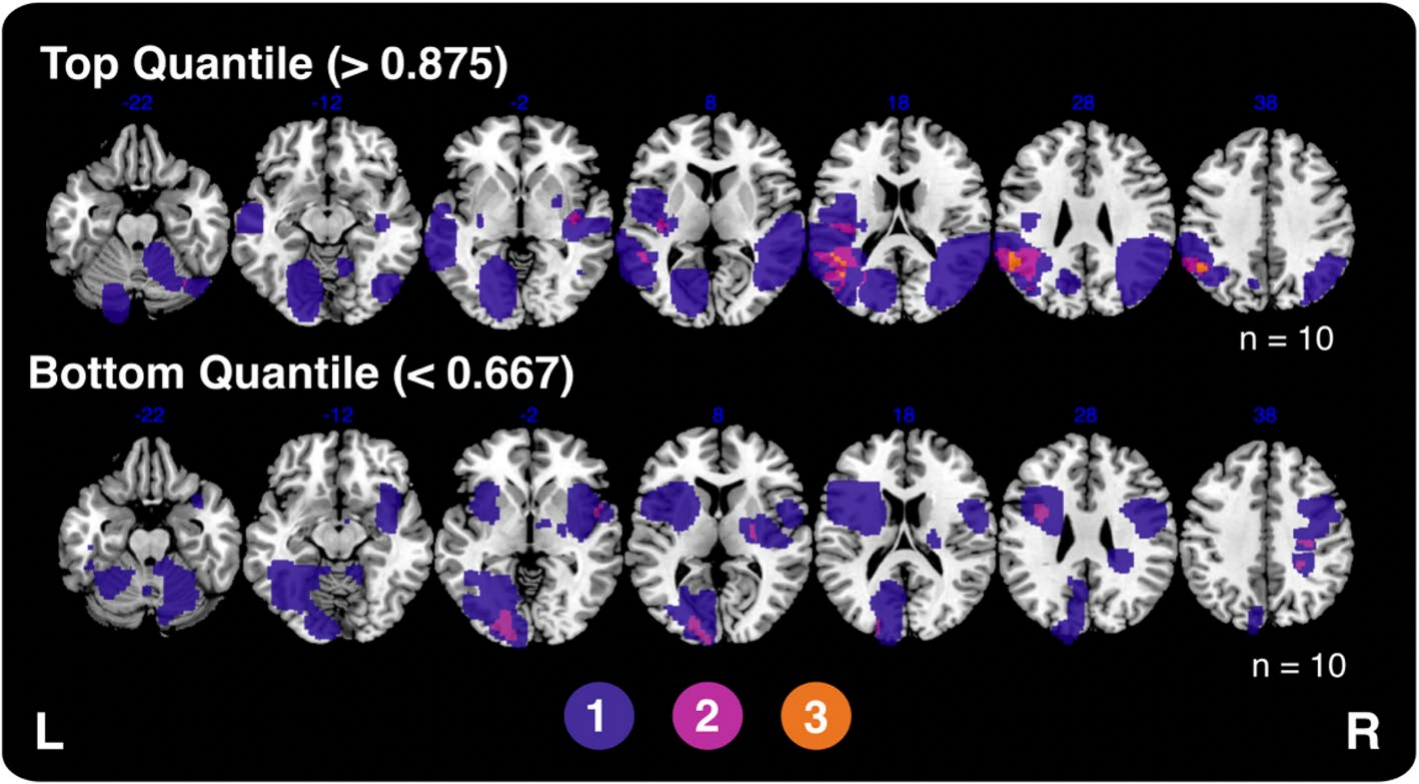
A comparison of the lesion overlays across patients committing the highest and lowest proportion of Category B errors on the Hayling test. Colour represents number of overlapping lesions. MNI slices − 22 to 36 are visualised

Figure 7 presents an overlap of the lesions from patients who committed the highest and lowest proportions of Category B errors. Specifically, the top panel visualises the lesions from patients in the top quantile (proportion of Category B errors > 0.875, *n* = 10) whilst the bottom panel visualises lesions from the bottom quantile (proportion < 0.667, *n* = 10). Both patient groups exhibited a wide variance in lesion locations with the highest degree of overlap (*n* = 3) centred within the left supramarginal gyrus. Notably, the patients in the top quantile exhibited no frontal lesions.

**Fig. 8:**
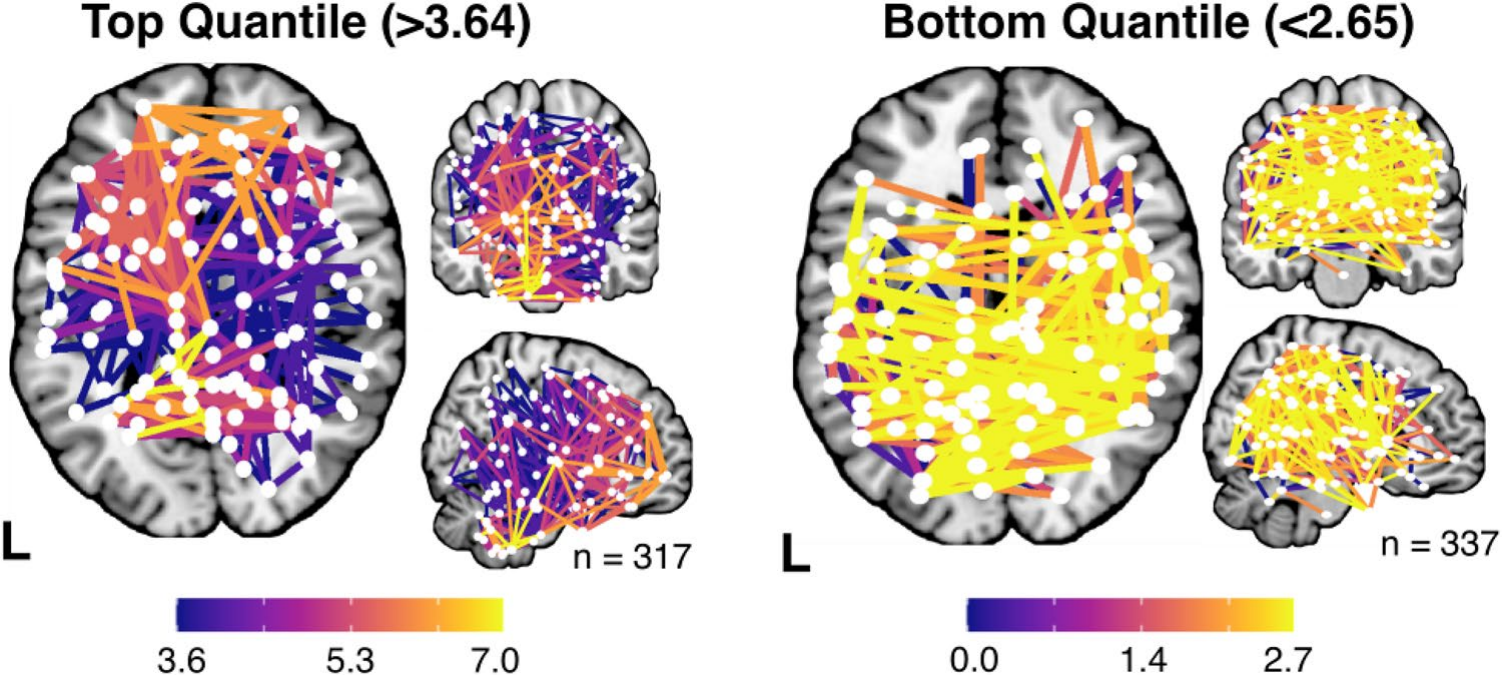
A visualisation of the network edges associated with comparatively high and low Category B error prevalence. Edge values represent the number of Category B errors committed in patients with damage to each edge divided by the number of times this edge was impacted in the full sample. All network nodes and edge locations are defined using the Schaefer-Yeo Atlas

Similar analyses were conducted to identify patterns of disconnection associated with the highest and lowest proportion of Category B Errors (Fig. 8). In total, 317 network edges were associated with high Category B error prevalence, whilst 337 edges were associated with comparatively low Category B error prevalence. Both these high and low prevalence edges spanned an expansive range of partially overlapping brain regions. However, the edges associated with high Category B prevalence appeared to involve slightly more anterior connections (e.g., frontal disconnection) as compared to the network associated with low Category B error prevalence (Fig. 8).

## Discussion

Overall, this project provides a novel investigation of Hayling test performance and strategy use within the stroke population. Although the Stroop test is commonly used to assess initiation and inhibition cognitive abilities, the results of this study strongly suggest that this task taps behaviourally and neuroanatomically distinct functions than those assessed in the Hayling test. Behavioural metrics derived from the Hayling test were found to provide detailed insight into abnormal patterns of both general performance impairment and strategy use in stroke survivors. These findings are important when considered in the context of clinical practice, as they highlight potential avenues for improving detection and diagnosis of common post-stroke cognitive impairments.

The findings of this study are in line with previous research suggesting that the Stroop and Hayling tests likely tap dissociable components of executive functions (Cipolotti et al. 2016). This conclusion is supported by both the behavioural and anatomical findings of this study. First, stroke patients were found to exhibit qualitative performance differences across Stroop and Hayling Test metrics intended to tap similar functions. Only minimal overlap was present between the specific patients exhibiting abnormal performance on analogous Stroop and Hayling test measures. While Stroop test metrics were found to have relatively high specificity to abnormal performance (specificity range = 75.0–90.5), this test was found to have very low sensitivity compared to analogous Hayling measures (sensitivity range = 10.0–66.6). This difference cannot be explained as a function of task difficulty, as performance on analogous Stroop and Hayling interference and inhibition measures were not found to be significantly correlated. In terms of neural correlates, only minimal overlap was present between the network-level correlates of analogous Stroop and Hayling test metrics. The highest degree of overlap was present between Stroop Dot Time and Hayling Initiation Time metrics, which tap initiation, with 6 (6.97%) of the 86 implicated network edges being common across both conditions. Overall, the findings of this investigation strongly suggest that the functions tapped by the Stroop and Hayling test are both behaviourally and anatomically dissociable.

There are several potential explanations for this dissociation. First, past research has suggested that it is possible for patients to complete the Stroop test without relying on the initiation/inhibition executive functions this test is designed to assess (Stuss and Alexander 2007). This is because participants can reduce interference from incongruent colour words by adopting strategies in which they do not read the full word whilst naming ink colours. This strategy may be more commonly adopted in the stroke population, as comorbid visual, spatial attention, language, or acquired dyslexia deficits would be expected to reduce the degree to which word stimuli are “automatically” read (Coslett and Turkeltaub 2016; Leff and Starrfelt 2014; Vallar et al. 2010). This potential confound may help explain the documented disparity between performance on purportedly analogous Stroop and Hayling test metrics. Additionally, Stroop test performance was found to vary widely in this study’s sample of healthy ageing controls, with many controls committing more errors than what was average within the patient sample. This is in line with previous work documenting declines in initiation/inhibition abilities within healthy ageing populations (Cervera-Crespo and González-Alvarez 2017; Gibson et al. 2018; West and Alain 2000). However, this high control variability does suggest that the Stroop test may not adequately be able to distinguish between age-related performance decrements and stroke-specific cognitive impairment. These implications are critically important when considered in the context of current clinical practice.

Variations of classical Stroop tasks are commonly used as screening tools for inhibition/initiation impairments within stroke survivors (Troyer et al. 2006). The findings of this study are critically important when considered in the context of this practice, as they suggest that abnormal performance on the Stroop test may not serve as an effective method for detecting these impairments in the stroke population. First, healthy ageing controls and stroke patients were not found to perform significantly differently on the Stroop colour-word task accuracy and interference scores. These metrics are the key measures expected to differ between clinical and control populations (Troyer et al. 2006). This lack of significant difference is likely due to the high score variance present within the control population coupled with the fact that not all stroke patients would be expected to perform abnormally on this task. This lack of difference alone does not undermine the validity of the Stroop Test but does suggest that cut-offs derived from this variable control performance may lack sensitivity to detect abnormal performance relative to other measures. This implication is supported by this study as only 5 patients met impairment criteria on Stroop-Colour Word Interference Scores, versus 14 on the analogous Hayling Response Time difference measure. This finding implies that more sensitive and targeted assessments are needed to detect inhibition/initiation deficits with high sensitivity in the stroke population.

The findings of this project suggest that the Hayling test may offer an effective alternative screening method for initiation and inhibition deficits in the stroke population. First, Hayling test metrics were found to be significantly different across stroke patient and control populations. Specifically, stroke survivors exhibited worse performance on Hayling Overall Scaled Scores, Global Error Scores, and Inhibition Accuracy versus control subjects. This is important when compared to the Stroop Test in which the only metric which was significantly different between controls and patients was the baseline dot-colour naming response time measure. In line with this, more patients were categorized as demonstrating abnormal performance on Hayling test measures relative to Stroop test measures, suggesting a comparative increase in screening sensitivity. In addition to this improved general sensitivity to potential impairment, Hayling strategy-use variables highlight additional differences between patient and control populations.

As found in previous studies, stroke participants committed more Hayling test suppression errors than controls (Laakso et al. 2019; Nijsse et al. 2019). However, this is the first investigation to explore Hayling task strategy use in stroke survivors. Previous work has explored differences in general strategy formation skills in stroke patients (e.g., Laakso et al. 2019), but this study did not quantify how differences in strategy formation may impact performance on the Hayling task. This analysis adds to this existing literature by providing novel insight into strategy differences underlying normal and abnormal Hayling test performance. First, a significantly higher proportion of control participant responses involved using a strategy to facilitate a correct response. Specifically, controls were significantly more likely to use strategies involving reporting visual items (See Table 3). While no other statistically significant group-level strategy-use differences emerged between patients and controls, strategy-use analyses facilitated the identification of individual patients exhibiting abnormal response types across a range of detailed, response-type metrics.

Notably, different Hayling test strategy-use measures were found to be associated with distinct network-level neural correlates. Specifically, correct responses involving reporting visual objects, visual objects related to previous responses, and other (undefined) strategy use were each significantly associated with non-overlapping patterns of network disconnection. Errors involving reporting responses semantically related to the sentence or bizarre errors were also found to be related to significant and distinct patterns of disconnection. These identified network-level correlates were found to be relatively restricted with comparatively few significantly involved nodes compared to general task metrics. However, these results did reach statistical significance following very strict corrections for lesion volume and multiple comparisons. Future, targeted studies are needed to fully quantify the patterns of disconnection associated with different Hayling test strategies and to interpret the functional significance of the identified network correlates. However, the present investigation is important in that it provides preliminary evidence that these different behavioural patterns are linked to distinct patterns of dysconnectivity. This is an important finding as it suggests that differences in strategy use are not just a result of patients preferred approaches but instead may be linked to distinct patterns of stroke-specific disconnection. This finding emphasizes the added utility of employing the Hayling Test in stroke populations, as it provides insight into what strategies patients are employing as well as assessing general initiation and suppression abilities.

This information can be used to direct more in-depth neuropsychological assessments and to detect abnormal strategy use patterns even in the absence of overall Hayling test impairment. The ability to generate and implement a strategy is crucial for rehabilitation to compensate for weaknesses and to facilitate the successful completion of everyday activities. Strategy training has been shown to be effective at overcoming suppression failures (Robinson et al. 2016), as well as multitasking (Rand et al. 2009) and cognitive flexibility and disability in stroke (Skidmore et al. 2015). We propose that analysis of strategy generation via the Hayling Test not only provides clinicians information regarding another aspect of executive functioning, but also provides information to support rehabilitation planning. That is, it can provide an indication of an individual’s capacity to problem solve and compensate for deficits. In this case, strategy generation and use appear to assist with overcoming difficulties with inhibitory control, which is important for goal-directed behaviour in daily life.

Notably, no significant network level correlates of Hayling Test Category B errors were identified in this study. This finding is surprising when considered in the context of previous functional imaging studies which have suggested that suppression abilities (as quantified by the Hayling test) are related to activity within a network of left prefrontal areas (Collette et al. 2001). There are several reasons why these previous findings were not replicated in this study. First, functional imaging is able to identify correlates involved in cognitive processes but cannot clearly distinguish whether these correlates are merely involved with or are necessary for the cognitive function of interest. It is possible that documented activation of left fronto-temporal areas may be related to the language component of the Hayling test (e.g., verbalising responses) rather than to cognitive inhibition functions. Qualitative analysis of Hayling test and lesion data suggest that disconnection of frontal network edges is associated with a disproportionately high prevalence of Category B Hayling errors, but further data is needed before this implication can be either confidently supported or refuted. It is also possible that this negative finding is related to variability in network-level statistical power, as the probability of committing network-edge level false negative detection varies as a function of how many patients have damage at each specific edge (Griffis et al. 2021).

Finally, it is possible that the null result produced by this analysis indicates that a wide range of lesions (and potentially underlying mechanisms) may modulate the occurrence of Hayling Category B Errors. In terms of lesions, the diversity in lesion sites associated with the occurrence of Hayling Category B errors is evidenced by Fig. 7. Contrary to expectations, many patients with posterior lesions were found to commit the highest proportion of Category B errors on the Hayling. This may indicate that critical networks underlying suppression ability may be disrupted at many, spatially distinct locations or that lesion location is not the only factor which modulates the occurrence of Hayling Category B errors. Past work has found that general measures of cortical atrophy and white matter integrity act as more effective predictors of executive function impairment than lesion-location metrics (Hobden et al. 2022). It is possible that a similar relationship may be present between pre-morbid atrophy/white matter integrity and Hayling test performance. Additional research is needed to explore each of these possibilities in detail and to further fundamental insight into the neural correlates underlying error commission on the Hayling test.

This novel demonstration of the Hayling Test within the stroke population suggests that this task represents an effective alternative measure which can detect initiation and inhibition deficits. The findings of this study are in line with past research indicating that the Stroop and Hayling Tests assess behaviourally and anatomically dissociable components of executive function. Behavioural metrics derived from the Hayling Test were found provide detailed insight into abnormal patterns of both general performance impairment and strategy use in stroke survivors and were able to link these patterns to distinct neural correlates. These findings suggest that the Hayling Test can be employed in acute stroke settings to provide a detailed and practical screen of initiation, inhibition and strategy use abilities in stroke survivors.

### Limitations

Executive dysfunction is a highly complex disorder and any one test in isolation is insufficient to fully characterise the pattern of impairment present in a patient. The results of this study suggest that the Hayling Test may serve as an effective first-line screen for initiation/inhibition impairment, but in-depth cognitive assessment is needed to fully characterise behaviour. First-line neuropsychological screens are important and practical clinical tools which are compatible with the time and resource limitations of real-world clinical environments (Moore et al. 2022). It, therefore, remains important to improve the efficiency of this first-line screening by identifying the tools which can provide the most detailed information within a short testing period.

Importantly, the Hayling Test may not be a suitable screen for all stroke survivors. A high percentage of the stroke population exhibit either language comprehension or production deficits which may preclude assessing initiation/inhibition performance on this language-dependent measure (Demeyere et al. 2015, 2020), although constraining an auditory context (i.e., sentence completion) has been found to facilitate production (Berndt et al. 2002; Robinson et al. 2005). Nevertheless, future studies can aim to develop and validate novel initiation/inhibition screens which are not dependent on language and can therefore be used in a greater portion of the stroke population.

The network-level analyses employed in this investigation use normative tractography atlases which may not exactly map onto the connectivity structure of the patients included in this analysis (Gleichgerrcht et al. 2017; Griffis et al. 2021). Similarly, number of disconnected streamlines can be unrelated to the strength of connectivity between regions (Fox 2018; Griffis et al. 2021). Despite these potential limitations, past studies employing similar normative atlas-based tractography atlases have agreed well with studies employing in-vivo tractography approaches.

Finally, past work has suggested that the non-random spatial distributions of stroke lesions may yield results which are displaced relative to the true underlying neural correlates of behavioural deficits in voxel-level lesion mapping analyses (Mah et al. 2014; Moore et al. 2023a, b, c). It is plausible that this effect is present in network-level analyses as well but is unlikely to have significantly impacted the main conclusions drawn in this study. Importantly, the aim of this study is to identify preliminary evidence of connectivity profile differences related to select tasks rather than to quantify the exact anatomy of each considered deficit. Future investigations can aim to expand on these findings by employing in-vivo tractography methods to improve fundamental understanding of the anatomy of suppression and initiation functions.

## Conclusion

Overall, this project provides a novel investigation of Hayling Test performance and strategy use within the stroke population. The results of this study strongly suggest that the Stroop and Hayling tests measure functions which are both behaviourally and neuroanatomically distinct. The Hayling Test was found to provide detailed insight into abnormal patterns of both general performance impairment and strategy use in stroke survivors. This novel demonstration of the Hayling Test within the stroke population suggests that this task represents an effective alternative measure which can detect initiation and inhibition deficits. These findings are important when considered in the context of clinical practice, as they highlight potential avenues for improving detection of common and debilitating post-stroke cognitive impairments and informing management and rehabilitation.

## Data Availability

All anonymised behavioural data, binarized lesion files, and analysis code have been made openly available on the Open Science Framework (https://osf.io/mbsd4/). All data which have not been made openly available due to copyright (e.g., test materials) and/or patient anonymity concerns (e.g., brain scans) are available on request from the authors or from the copyright holders where relevant.
